# The medieval Mongolian roots of Y-chromosomal lineages from South Kazakhstan

**DOI:** 10.1186/s12863-020-00897-5

**Published:** 2020-10-22

**Authors:** Maxat Zhabagin, Zhaxylyk Sabitov, Pavel Tarlykov, Inkar Tazhigulova, Zukhra Junissova, Dauren Yerezhepov, Rakhmetolla Akilzhanov, Elena Zholdybayeva, Lan-Hai Wei, Ainur Akilzhanova, Oleg Balanovsky, Elena Balanovska

**Affiliations:** 1 National Laboratory Astana, Nazarbayev University, Nur-Sultan, Kazakhstan; 2 National Center for Biotechnology, Nur-Sultan, Kazakhstan; 3 L.N. Gumilyov Eurasian National University, Nur-Sultan, Kazakhstan; 4 Young Researchers Alliance, Nur-Sultan, Republic of Kazakhstan; 5 Forensic Science Center of the Ministry of Justice of the Republic of Kazakhstan, Nur-Sultan, Kazakhstan; 6 Research Institute of Archeology named after K.A. Akishev, Nur-Sultan, Republic of Kazakhstan; 7 S. Toraighyrov Pavlodar State University, Pavlodar, Kazakhstan; 8 B&R International Joint Laboratory for Eurasian Anthropology, Fudan University, Shanghai, China; 9 Department of Anthropology and Ethnology, Institute of Anthropology, Xiamen University, Xiamen, China; 10 Vavilov Institute for General Genetics, Russian Academy of Sciences, Moscow, Russia; 11 Research Centre for Medical Genetics, Moscow, Russia; 12 Biobank of North Eurasia, Moscow, Russia

**Keywords:** Human genetics, Y-chromosome, Short tandem repeat, Single nucleotide polymorphism, Time to the most recent common ancestor, Kazakh, Mongol, Wusun

## Abstract

**Background:**

The majority of the Kazakhs from South Kazakhstan belongs to the 12 clans of the Senior Zhuz. According to traditional genealogy, nine of these clans have a common ancestor and constitute the Uissun tribe. There are three main hypotheses of the clans’ origin, namely, origin from early Wusuns, from Niru’un Mongols, or from Darligin Mongols. We genotyped 490 samples of South Kazakhs by 35 Y-chromosomal SNPs (single nucleotide polymorphism) and 17 STRs (short tandem repeat). Additionally, 133 samples from citizen science projects were included into the study.

**Results:**

We found that three Uissun clans have unique Y-chromosomal profiles, but the remaining six Uissun clans and one non-Uissun clan share a common paternal gene pool. They share a high frequency (> 40%) of the C2*-ST haplogroup (marked by the SNP F3796), which is associated with the early Niru’un Mongols. Phylogenetic analysis of this haplogroup carried out on 743 individuals from 25 populations of Eurasia has revealed a set of haplotype clusters, three of which contain the Uissun haplotypes. The demographic expansion of these clusters dates back to the 13-fourteenth century, coinciding with the time of the Uissun’s ancestor Maiky-biy known from historical sources. In addition, it coincides with the expansion period of the Mongol Empire in the Late Middle Ages. A comparison of the results with published aDNA (ancient deoxyribonucleic acid) data and modern Y haplogroups frequencies suggest an origin of Uissuns from Niru’un Mongols rather than from Wusuns or Darligin Mongols.

**Conclusions:**

The Y-chromosomal variation in South Kazakh clans indicates their common origin in 13th–14th centuries AD, in agreement with the traditional genealogy. Though genetically there were at least three ancestral lineages instead of the traditional single ancestor. The majority of the Y-chromosomal lineages of South Kazakhstan was brought by the migration of the population related to the medieval Niru’un Mongols.

## Background

Patrilineal populations tend to have deep and extensive paternal genealogies. Such populations from the Eurasian steppe are traditionally divided into many descent groups (tribes, clans, lineages). Since clan affiliation is paternally inherited along with the Y chromosome, a joint comprehensive study of the clan structure and the Y chromosome variation increases the depth and accuracy of a reconstruction of the demographic history.

**Fig. 1:**
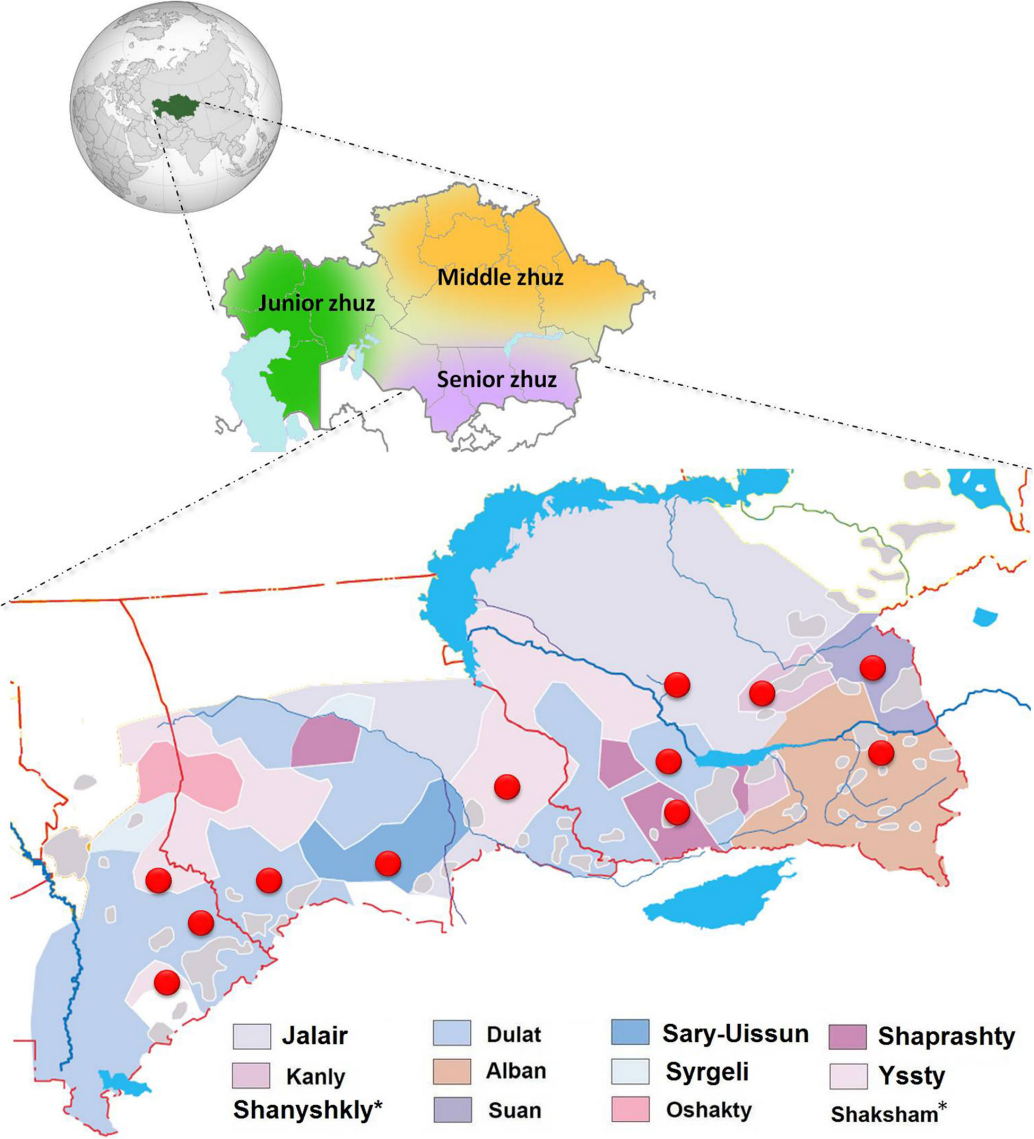
Distribution of the Senior Zhuz and the studied clans (modified from [1]). *Note: Shanyshkly and Shaksham are very small populations and dispersed settled, that is why the area is not highlighted in color

Kazakhs have one of the largest clan structures in the Eurasian steppe. Kazakh clans are structured into three main socio-territorial groups called Senior, Middle, and Junior *Zhuzes* (Fig. 1). Twelve clans of the Senior Zhuz (Additional file 1) mainly reside in the South Kazakhstan. According to the traditional genealogy of the Kazakhs, also known as *Shezhire*, nine out of 12 clans share a common ancestor known as Maiky-biy. Historical sources mention that he led the western part of the Golden Horde under Batu Khan, the grandson of Genghis Khan. These nine clans altogether form the Uissun tribe [2]. The three remaining clans (Jalair, Kanly, and Shanyshkly) have their own ancestors and are considered as genealogically unrelated to each other and to Uissun clans [3].

There are three main hypotheses of the origin of the Uissun tribe. The first one is the origin from the early Wusuns [4], people of Iranian or Tocharian origin, who lived in the Tarim river basin. This area is adjacent to the location of the current residence of the Uissuns [5]. The other two versions imply a more distant migration from Mongolia [6]. According to the genealogy, Maiky-biy either belongs to the clan of Ushin being a part of the Darligin Mongols [7], or to the clan of Baarin being a part the Niru’un Mongols [8].

Recently, Ashirbekov and coauthors analyzed the Y-chromosomal variation of South Kazakhs [9]. However, this study describes the frequencies of main Y chromosomal haplogroups only, while detailed analysis of their branches and STR haplotypes has not been published. Detailed subclades of the Y chromosome are known only for one part of the Alban clan living in the Xinjiang Uygur District of China. For example, a subclade of haplogroup C2-F3796 was found with a high frequency of 44% [10]. Therefore, the present study is devoted to the genetic study of the Senior Zhuz clans based on in-depth phylogenetic analysis based on 35 SNPs and 17 STRs of the Y chromosome. The purpose of the work is not only to characterize the fine structure of the gene pool of the populations of South Kazakhstan but also to identify which out of the three versions of their origin finds genetic verification.

## Results and discussion

### Paternal genetic portraits of south Kazakh clans

**Fig. 2:**
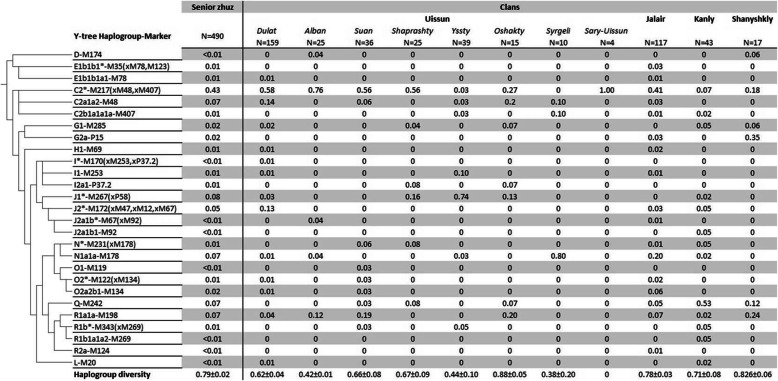
Frequencies of Y-chromosomal haplogroups in Kazakh clans of the Senior Zhuz

We genotyped 35 Y-SNP and 17 Y-STRs markers in 490 individuals, representing 11 clans of South Kazakhstan – eight clans of Uissun tribe and three clans which belong to Senior Zhuz but are not considered as members of the Uissun tribe. Twenty-seven Y-chromosomal haplogroups were identified in this sample (Fig. 2, Additional file 2).

More than half of the Y-chromosomal gene pool (51%) in South Kazakhs (Senior Zhuz) is characterized by three clades of haplogroup C2 (Fig. 2). One-third (34%) of the gene pool is shaped by the haplogroups J-M172 (13%), N1a1a-M178 (7%), Q-M242 (7%), and R1a1a-M198 (7%). Though haplogroups are not frequent in South Kazakhstan in general, they become predominant within the specific clans (Fig. 2).

For most clans of South Kazakhstan, the haplogroup C2-M217 (xM48, M407) is a major one. Among the eight clans of the Uissun tribe, C2-M217 is the most frequent for six of them (Dulat, Alban, Suan, Shaprashty, Oshakty, and Sary-Uissun). It is also the most frequent for the non-Uissun clan of Jalair. For the two remaining Uissun clans, the major haplogroups are N1a1a-M178 (80% of the Syrgeli clan) and J1*-M267(xP58) (74% of the Yssty clan). For the two remaining non-Uissun clans, the major haplogroups are Q-M242 (53% of the Kanly clan) and G2-P15 (35% of the Shanyshkly clan).

Though sample sizes for the four clans are small (*N* < 20) the samples for the remaining clans are representative (*N* = 63 on average); therefore, there is strong evidence for the prevalence of haplogroup C2-M217(xM48, M407) among Kazakhs of the Senior Zhuz. It was reported, that C2* is the major haplogroup (88%) in the Sary-Uissun clan [9], and the same haplogroup is major for the Alban clan of the Xinjiang Uyghur District of China (44%) [10]. Interestingly, the evidence that the Shanyshkly clan are contradictive: according to citizen science projects, a high frequency of the haplogroup C2* (Additional file 3) was detected; data from the present study reports only 18% of C2*, while the most common haplogroup is G2-P15 (36%).

### Genetic structuring of the paternal gene pool of Uissun

The genetic portraits of the three Uissun clans (Yssty, Syrgeli, and Oshakty) are very specific (Fig. 2) in contrast to the genealogy, suggesting the origin of all nine Uissun clans (Additional file 1) from a common ancestor named Maiky-biy. The genetically distinct origin is also confirmed by AMOVA analysis. In this analysis, we either considered seven Uissun clans as independent branches or left separate only three genetically specific clans grouping the remained four into the “core Uissun” population. This structure of the Uissun clans, consisting of four groups, turned out to be more efficient (F_ST_ = 0.35) than without substructuring into groups (F_ST_ = 0.24) (Additional file 4). A similar result was observed using multidimensional methods of statistical analysis (MDS and PCA). A single cluster for seven clans (Sary-Uissun, Dulat, Alban, Suan, Shaprashty, Oshakty and Jalair) is highlighted on the MDS chart (Additional file 5). On the PCA plot, five clans (Sary-Uissun, Dulat, Alban, Suan, and Shaprashty) form a cluster along the first component axis, which is approached by the Oshakty clan. The Jalair clan moves away along the second component axis (Additional file 6).

### Phylogenetic analysis of haplogroup С2*-F3796

We performed the detailed phylogenetic analysis of the most frequent haplogroup among the Uissuns – C2*-ST (40%). This haplogroup, also known as Star Cluster (ST), is clearly distinguished within M217(xM48, M407) by STR haplotypes. It corresponds to the subclade marked by the SNP F3796 [10]. This lineage had spread rapidly over the steppe in Eurasia during the conquests of the Mongol Empire. It has been presumably associated with the haplotype of Genghis Khan or his relatives [11]. The highest frequencies of the C2*-ST were found in Kazakhs from the Kerey clan of the Middle Zhuz (77%) [12], Buryats from the Bargut clan (46%) [10], Hazaras (38%) [13]; Uzbeks from Afghanistan (35%) [14], and Mongols (35%) [15]. The highest haplotype diversity in C2*-ST is specific for the Mongols (HD = 0.91) and Uzbeks (HD = 0.95), and the lowest diversity was found for the Kazakhs, both for the Uissuns (HD = 0.86) and other tribal groups (HD = 0.84).

**Fig. 3:**
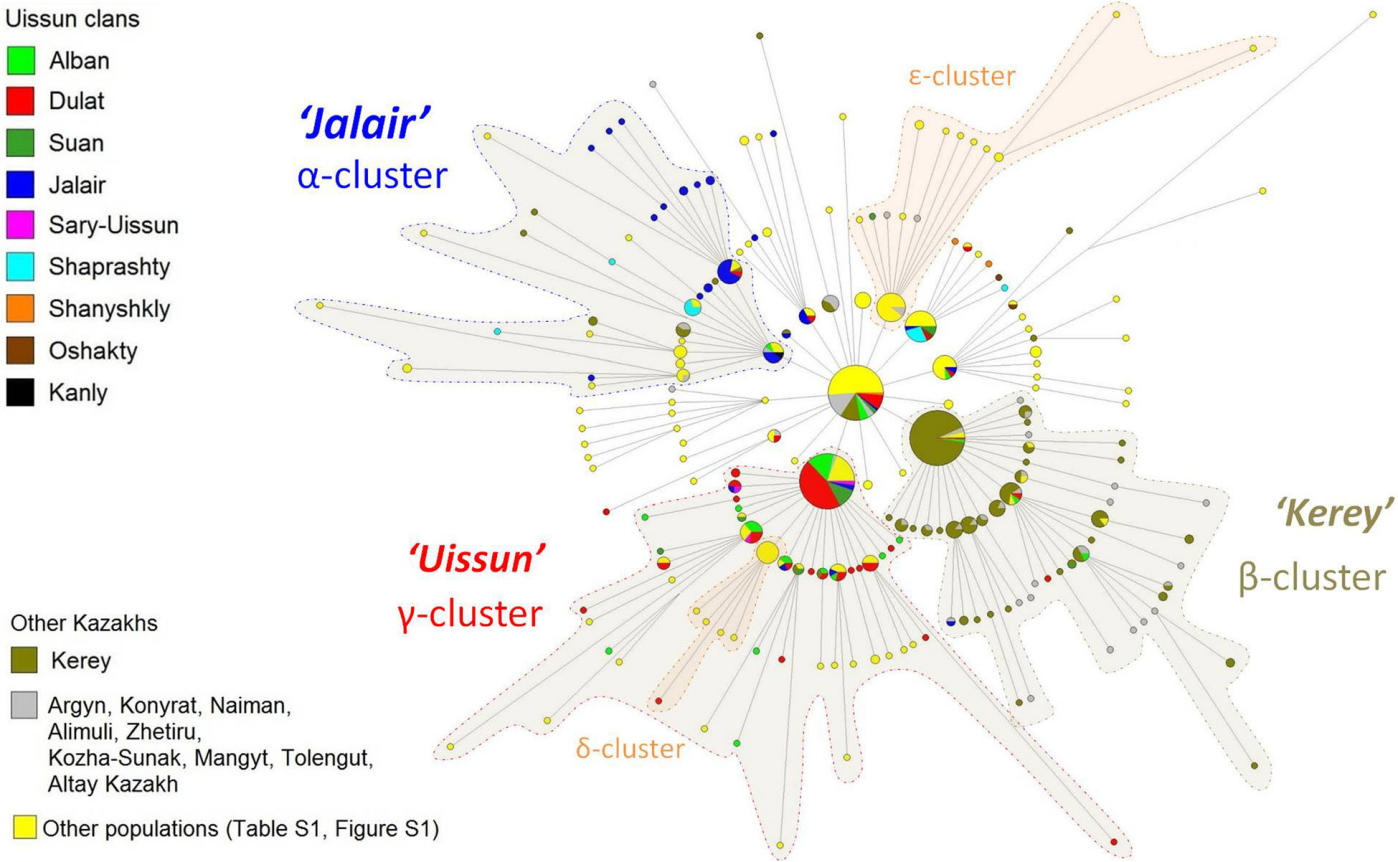
Y-STR network of Kazakh - C2*- ST based on 15 Y-STRs

The phylogenetic network of haplotypes within the haplogroup C2*-ST (C-F3796) was constructed using 15 STR loci of the Y chromosome according to data on 743 individuals from 25 populations of Eurasia (novel samples, *N* = 194, Additional file 2; previously published, *N* = 549, Additional file 7). Additional file 8 presents the haplotypes of the Eurasian ethnic groups, while Fig. 2 highlights the haplotypes of the Kazakh clans on the same network (other ethnic groups are shown in yellow). Five distinct clusters are clearly visible on the network (Fig. 3). Majority of the samples from South Kazakhstan (the Senior Zhuz) were included into the two newly identified C2*-ST (Additional file 9) subclusters: α-cluster (*N* = 44) and γ-cluster (*N* = 122), and only a few samples (*N* = 9) entered the β-cluster (dating 701 CI 95% 909–493 years), previously identified for the Kerey clan of the Middle Zhuz. The α-cluster (dated 746 CI 95% 1104–388 years) was mainly composed of the the (non-Uissun) Jalair clan members. The γ-cluster (dating 742 CI 95% 916–568 years), which we called “Uissun” cluster, mainly included representatives of the four Uissun clans, namely, Dulat, Alban, Suan, and Sary-Uissun (Fig. 3). Among the two Hazara subclusters, the δ-cluster originates from the Uissun γ-cluster, while the ε-cluster is derived from the common C2*ST founder and includes, in addition to the Hazaras, a few Uzbek haplotypes.

### Comparison TMRCA by SNP and STR of Y-chromosome

The TMRCA (time to the most recent common ancestor) of the C2*-ST cluster (Fig. 3) based on Y-STR profiles is estimated to be 1544 CI 95% 1968–1120 years. In contrast, TMRCA based on SNPs from sequencing data of 17 Y-chromosome samples [10] is ~ 2600 years ago. This dating coincides with citizen science data from 15 Y-chromosome sequences (the TMRCA of the C2*-ST cluster based on SNPs is ~ 2500 years (TMRCA CI 95% 3200–1850 ypb) (www.yfull.com)). What is the reason for a significant discrepancy between 1500 years estimated by STRs and 2500 years resulted from SNP data? It may be explained by the incomplete mapping of STR clusters and the topology of the phylogenetic tree, since different SNP subclades may have the same STR haplotypes, as shown in Additional file 9. For α and β clusters, which included samples from South Kazakhstan, we also compared the dates obtained by SNP and STR data. The α-cluster TMRCA based on SNPs (four Y chromosomes sequenced) turned out to be ~ 750 years (TMRCA CI 95% 1050–400 ybp) (subclade Y12782, a sample from the Dulat clan [16], www.yfull.com), which is very close to STR dating (746 CI 95% 1104–388 years). The TMRCA of the β-cluster based on SNPs from three sequenced Y chromosomes (~ 650 years ago (TMRCA CI 95% 346–982 ybp), C2-F8949 subclade, previously identified as the only Kazakh subclade on the C2*-ST network [10]) was also very close to the dating by STRs (701 CI 95% 909–493 years). Unfortunately, there are no sequences of extended sections of Y-chromosomes for the γ-cluster, and the SNP marker defining this subclade has not yet been determined. The TMRCA of the γ cluster based on STRs is 742 CI 95% 916–568 years.

The nearby coincidence of ages of all three clusters suggests the rapid population growth of Kazakh clans in 13-14th centuries. It nicely coincides with the expansion period of the Mongol Empire. It is important to note that the TMRCA of the “Uissun” γ-cluster coincides with the lifetime of the proposed Uissun’s ancestor and Genghis Khan’s ally Maiky-biy (thirteenth century).

### Whose descendants are the clans of South Kazakhstan?

We found that C2-F3796 subclade of haplogroup C2*-ST is the most common in the population of South Kazakhstan. In this sense C2*-ST is a key to decipher direct paternal ancestor of the Senior Zhuz clans. Moreover, according to historical studies [2], the lifetime of the legendary ancestor of the Uissuns (the main population group of South Kazakhstan) coincides with TMRCA of the Uissun cluster.

The oldest known specimen of this lineage (subclade C2-Y4580*) originated from the Mongolian-Buddhist burial of Ulus Dzhuchi (700 years ago) in Central Kazakhstan (Ulytau, Karasauyr burial ground [17]). It is closely related to the Uissun haplogroup C2*-ST.

The only sample of the Wusun culture studied to date (burial Turgen-2, Semirechye, Kazakhstan) belongs to the haplogroup R1a1a-Z93(xZ94) (subclade R1a1a-Y41571) [17]. Other ancient specimens from the Tarim Basin where Wusun lived also belonged to the haplogroup R1a1 [18]. In contrast, all previously studied Kazakh samples belonged to another branch of R1a, namely R1a1a-Z94 (subclade Z2125) [16, 19]. In general, R1a is not frequent among Uissun (6% only), therefore, paternal lineages of the Uissuns likely originated from the early Mongols populations rather than from the Wusun.

According to The Secret History of the Mongols, the early Mongols were divided into Niru’un and Darligin Mongols [6]. Which one of them is the ancestor of the Uissuns? The only successor clan of the Darligin Mongols which has been genetically studied is Konyrat (Kungirat) [6, 20]. The haplogroup C2-M407 is present at high frequency (86%) in Konyrat (Additional file 10), but not in the Uissuns. According to genealogy (Additional file 11), not only the Uissuns but also the Shanyshkly clan of the Senior Zhuz are the descendants of the Niru’un Mongols with dominant C2*-ST haplogroup. In addition, C2*-ST is identified by citizen scientists in several genealogical lineages of the Niru’uns (Keneges, Manghit and Katagan) [21], and among the Hazaras which are considered to be direct descendants of the Niru’un Mongols [10]. As a result, we suggest the origin of the Y-chromosomal lineages of the main populations of South Kazakhstan from the Niru’un Mongols.

### Analysis of the downstream SNPs within С2*-ST in south Kazakh clans

A subset of samples (*N* = 71) has been genotyped by the high resolution SNPs within the haplogroup C2*-ST. First, we genotyped F3796 and F8951, defining two parallel clades, in all 71 samples (Additional file 2). We identified 70 F3796-positive samples and one F8951-positive sample, in perfect agreement with what was predicted from the STR-profiles. New, we genotyped all F3796-positive samples for the 8 downstream markers, reflecting the topology of F3796. This clade (Additional file 9) includes three subclades: F3960 and SK1072 are typical for Mongolic-speaking populations, while the third subclade includes both, Mongolian branch (F9747) and West Central Asian branch (F5481) [10, 22]. This West Central Asian branch includes at least five subbranches: SK1076, F8949, F9033, F11165, Y12782. Our results indicated, that the subbranch Y12782 is most frequent among South Kazakh’ F3796 samples (76%), particularly among Alban, Dulat, and Suan clans. The absolute frequency of this Y12782 subbranch in Uissun tribe is 31%. As for Zhalaiir, most of them (82%) belong to the F5481 branch, but not to the any of its reported subbranches.

## Conclusions

We presented the Y-chromosomal profiles of the almost every clan from South Kazakhstan (the historical area of the Senior Zhuz of Kazakh). The results indicated the genetic similarity of the six Uissun and one non-Uissun clans to each other, while the other four clans (two Uissun and two non-Uissun clans) have the specific paternal pools. Thus, the genetic data have not reproduced the traditional genealogy in all details; however, the genetic evidences were consistent with the common origin of the most clans from South Kazakhstan. Moreover, the significant part of the population originated from three founders which all lived about 700–800 years ago, in contrast to one founder, according to traditional genealogy. These three clusters were identified within the C2*-ST (C-F3796) haplogroup. The first cluster is typical for most Uissun clans, the second one is common for the non-Uissun clan Jalair of South Kazakhstan, and the third one is typical for the North Kazakhstan clan of Kerey, but also includes individual samples from South Kazakhstan. The predominance of the haplogroup C2*-ST in South Kazakhstan suggests the origin of the majority of Y-chromosomal lineages from the Niru’un Mongols.

The molecular genealogy of the male descendants of the Niru’un Mongols assumes a detailed study of all branches (Additional file 11) using sequencing of full Y-chromosomes in the future. In particular, SNP typing for Y12782 subclades (“Uissun” α-cluster), F8949 (“Kerey” β-cluster) and SNP subclade detection for the Jalair γ-cluster, as well as a comparison of the Uissuns with data on ancient DNA of proto-Mongols and early Wusun people on the whole-genome scale are needed.

In conclusion, our data do not confirm the hypothesis of the Uissun’s origin from the early Wusuns or from the Darligin Mongols [4, 7]. Instead, the genetic similarity of the paternal lineages of the population from South Kazakhstan and the early Niru’un Mongols has been proposed.

## Methods

Blood samples of the Kazakh male individuals were collected in Kazakhstan during field trips conducted in 2009, 2010, and 2014 following the rules of biobanking for population genetics [23]. Ethical approval was received from the Ethics Committee of the Research Centre for Medical Genetics (Moscow, Russia), National Center for Biotechnology and the National Laboratory Astana (Nur-Sultan, Kazakhstan). Eleven clans of South Kazakhstan were studied. In total 490 samples were collected with the written informed consent.

Genomic DNA extraction, genotyping, statistical analysis and median network analysis were done as described previously [20]. The 35 Y-chromosomal SNPs (M130, M217, M48, M407, M174, M35.1, M78, M123, M285, P15, M69, M170, M253, P37.2, M267, P58, M172, M47, M67, M12, M92, M20, M231, M178, M175, M119, M122, M134, M242, M207, M198, M343, M269, M124, M70) were genotyped using TaqMan assays on the 7900HT Real-Time PCR System. Haplogroups name were given according to the nomenclature of International Society of Genetic Genealogy (https://isogg.org/). Fragment analysis of 17 Y-STR loci was performed with the Y-filer PCR Amplification Kit (Life Technologies) on the ABI 3130xl and 3500xl genetic analyzers. Y-STR haplotypes of C2*-ST were identified based on the Wei paper [10]. A subset of Kazakh samples (*N* = 70) belonged to C2*-ST and one sample belonged to Daur Clade were genotyped by F3796 and F8951 SNPs. Subclades of C2*-ST were also genotyped by (F11899[synonym F9700], F1072, F117991[synonym F9747], F5481, F8949, F9266[synonym F9033], F11165, Y12782). Phylogenetic networks of Y-STR haplotypes were constructed using the Network 5 and Network Publisher software [24, 25], excluding DYS385a/b. Data on 549 samples belonged to C2*-ST haplogroup from 25 populations were used from previous studies (Additional file 7). The stability of the phylogenetic pattern has been investigated by random omitting 10% of samples and repeating the analysis; the same clusters have been identified in this experiment. Cluster ages were determined using the rho-statistic [26, 27]. The mutation rate of 2.1 × 10^− 3^ mutations per STR per generation was used [28]. The generation time was set to 30 years [29]. Additional data on 133 samples belonged to Senior *Zhuz* were collected from citizen science databases [30] and analyzed for discussion (Additional file 3). Nei’s genetic distances between clans were calculated using and the DJ software [31]. Multidimensional scaling, cluster analysis (Ward’s method), and principal component analysis were conducted using the Statistica v.7.1 software [32].

## Supplementary information

**Additional file 1: Text S1.** Genealogical description of the Senior Zhuz clans.

**Additional file 2: Table S1.** Y chromosome SNP and STR data of the Uissun tribe.

**Additional file 3: Table S2.** Y chromosome SNP and STR data of the Uissun tribe from citizen science.

**Additional file 4: Table S3.** Variation in Y-chromosomal haplogroup frequencies between Uissun clans.

**Additional file 5: Figure S1.** Genetic relationships of Great zhuz’s clans using Y-SNPs: Multidimensional scaling plot (MDS)

**Additional file 6: Figure S2.** Genetic relationships of Great zhuz’s clans using Y-SNPs: Principal component analysis (PCA) of the Uissun tribe

**Additional file 7: Table S4.** 15 Y-STR haplotypes of all C2*-ST samples from previous studies included in the network.

**Additional file 8: Figure S3.** Y-chromosome’s Network of C2*-ST based on 15 Y-STRs.

**Additional file 9: Figure S4.** Comparison between STR network and SNP phylogenetic tree of the Y chromosome.

**Additional file 10: Table S5.** Y-chromosome SNP and STR data of Konyrat tribe: update up to C2b1a3a-M407

**Additional file 11: Figure S5.** Genealogy of the Niru’un Mongols.

## Data Availability

All data generated or analysed during this study are included in this published article and its supplementary information files.

## Abbreviations

SNP: Single nucleotide polymorphism
STR: Short tandem repeat
aDNA: Ancient deoxyribonucleic acid
F_ST_: Fixation index
AMOVA: Analysis of molecular variance
MDS: Multidimensional scaling
PCA: Principal component analysis
HD: Haplotype diversity
TMRCA: Time to the most recent common ancestor
PCR: Polymerase chain reaction
