# Supplementary material for: The medieval Mongolian roots of Y-chromosomal lineages from South Kazakhstan
Source: BMC Genet. 2020 Oct 22;21(Suppl 1):87. doi: 10.1186/s12863-020-00897-5 (PMC7583311; doi:10.1186/s12863-020-00897-5)
Supplement: Supplementary file 1 — Additional file 1: Text S1. Genealogical description of the Senior Zhuz clans. [file 12863_2020_897_MOESM1_ESM.docx]

*Supplementary text 1*

**The medieval Mongolian roots of Y-chromosomal lineages from South Kazakhstan**

Maxat Zhabagin^1,2^*, Zhaxylyk Sabitov^3,4^, Pavel Tarlykov^2^, Inkar Tazhigulova5, Zukhra Junissova^6^, Dauren Yerezhepov^1^, Rakhmetolla Akilzhanov^7^, Zholdybayeva Elena^2^, Lan-hai Wei^8,9^, Ainur Akilzhanova^1^, Oleg Balanovsky^10,11,12^, Elena Balanovska^11,12^

^1^National Laboratory Astana, Nazarbayev University, Nur-Sultan, Kazakhstan

^2^National Center for Biotechnology, Nur-Sultan, Kazakhstan

^3^L.N. Gumilyov Eurasian National University, Nur-Sultan, Kazakhstan

^4^Young Researchers Alliance, Nur-Sultan 010000, Republic of Kazakhstan

^5^Forensic science center of the Ministry of Justice of the Republic of Kazakhstan, Nur-Sultan, Kazakhstan

^6^Research Institute of Archeology named after K.A. Akishev, Nur-Sultan, Republic of Kazakhstan

^7^S. Toraighyrov Pavlodar State University, Pavlodar, Kazakhstan

^8^B&R International Joint Laboratory for Eurasian Anthropology, Fudan University, Shanghai, China

^9^Department of Anthropology and Ethnology, Institute of Anthropology, Xiamen

University, Xiamen, China

^10^Vavilov Institute for General Genetics, Russian Academy of Sciences, Moscow, Russia

^11^Research Centre for Medical Genetics, Moscow, Russia

^12^Biobank of North Eurasia, Moscow, Russia

*Corresponding author’s e-mail: [mzhabagin@gmail.com](mailto:mzhabagin@gmail.com)

**Genealogical description of the Senior Zhuz** **clans**

by Zhaxylyk Sabitov, Zukhra Junissova and Maxat Zhabagin

The approximate number of Kazakhs of the senior zhuz at the end of the 19th century was about 700 thousand people [1]. The senior zhuz includes the following tribes: Uissun, Jalair, Kangly, Shanyshkly. The founder of the Uissun tribe was Uissun [2]. His son was Maiky-biy. Maiky-biy is a historical figure, adviser to Batu (grandson of Genghis Khan), who was the ruler of the Golden Horde (Ulus Jochi) in 1227-1255. Maiky-biy had a great-grandson, whose name was Karasha-biy. Karasha-biy had two sons: Baidibek and Baidaul [3]. Another descendant of Maiky-biy is Oisyl [4]. According to historical sources, Oisyl (Isa) lived in the first half of the XIV century during the reign of Uzbeg-khan (1313-1341) and Janibeg-khan (1342-1357). Oisyl's son was the son-in-law of Janibek Khan [5]. The descendants of Baidibek are representatives of the following tribes: Sary-Uissun, Dulat, Alban, Suan, Yssty, Shaprashty, Oshakty. Descendants of Baidaul are representatives of the Shaksham tribe. Oisyl descendants are representatives of the Syrgeli tribe.

Figure 1. Genealogy of the Uissun tribe**.**

**1. Sary-Uissun.** The Sary-Uissun tribe settled in the following territories: the left bank of the Ili River, the foothills of the Zailiysky Alatau, the right bank of the Talas River, along the banks of the Kuragaty River until it flows into the Chu River [1]. The number of the tribe at the end of the 19th century was 10 thousand people [1]. The "Uran" (battle exclamation) of the Sary-Uissun tribe is Baytok. Tamga (graphic sign of ownership) of this tribe is called "Abak-Ashamay" [2].


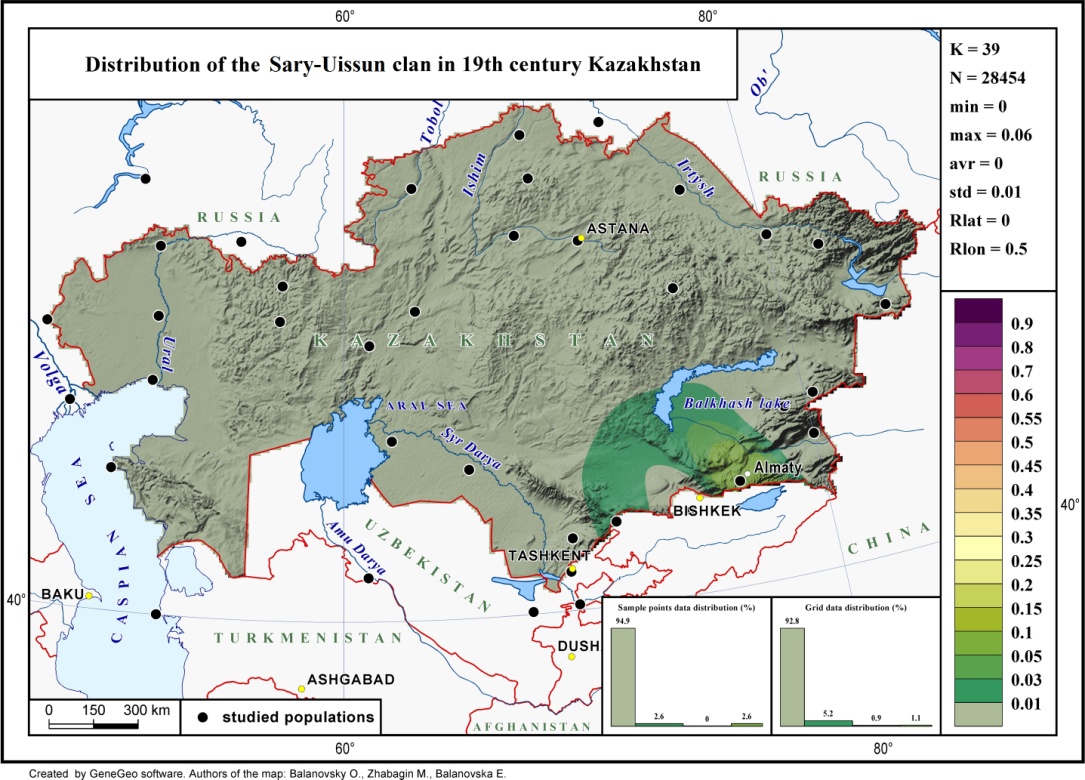


Figure 2. Map of the Sary-Uissun tribe resettlement [6].

Figure 3. Genealogy of the Sary-Uissun tribe.

**2. Dulat.** The Dulat tribe settled in the following territories: from the middle course of the Ili river to the Chu and Talas rivers, from the foothills of the Kyrgyz Alatau and Karatau to the middle course of the Syr Darya river, from the western part of the Tien Shan mountains to the middle course of the Chu river, and the southern coast of Lake Balkhash [1]. The number of the tribe at the end of the 19th century was 250 thousand people [1]. There are three versions of the origin of the Dulat tribe. According to the first point of view, the founder of this tribe was called Dulat. He was the son of Zharykshak, the grandson of Baidibek. That is, Dulat is part of the Uissun tribe. According to the second point of view, the Dulat are descendants of the Mongolian Duglat tribe, which was the ruling clan in Mogulistan. According to the third point of view, they come from the tribal union of Dulu, which existed in the 7th century in the Western Turkic Kaganate. The "Uran" of the Dulat tribe is Bakhtiar. Tamga of this tribe is called "Abak" [2].


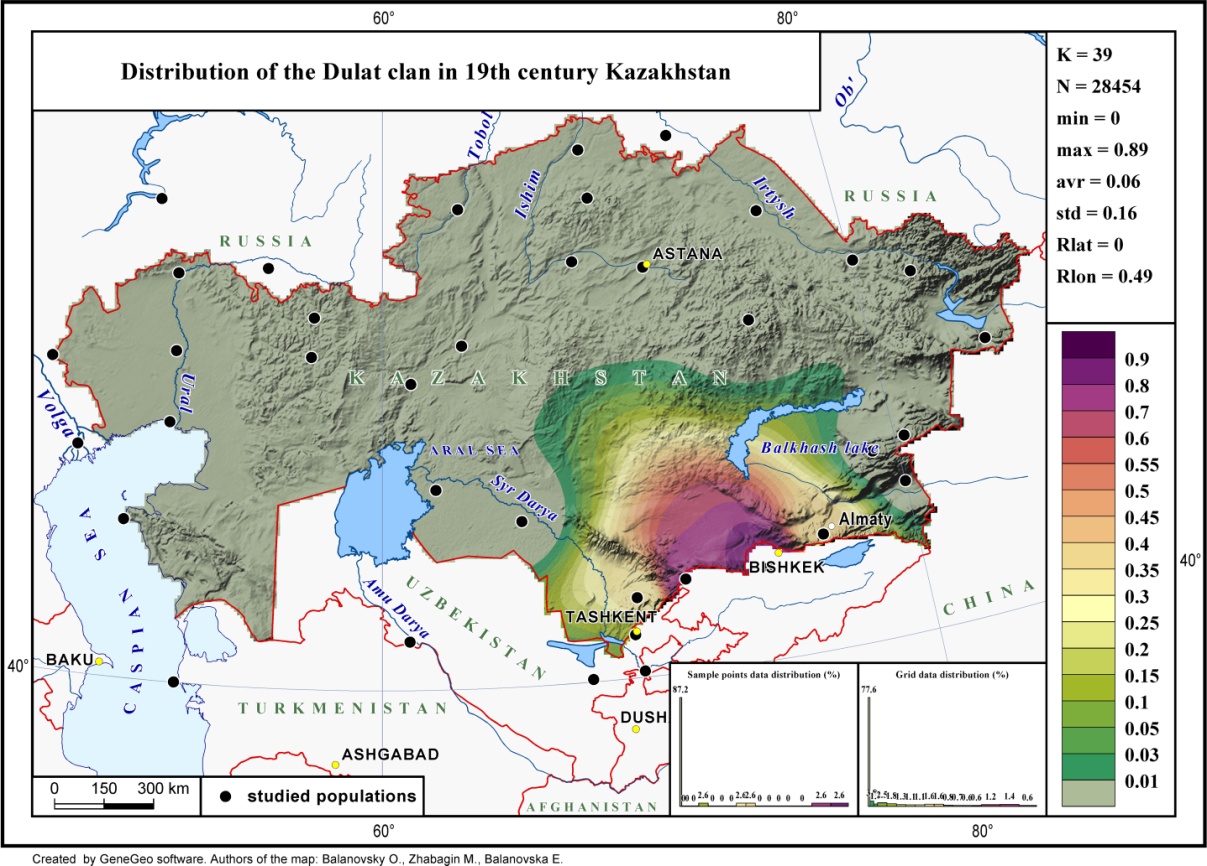


Figure 4. Map of the Dulat tribe resettlement [6].

Figure 5. Genealogy of the Dulat tribe.

**3. Alban.** The Alban tribe settled in Zhetysu (Semirechye) in the following territories:. from the southeastern slopes of the Altyn-Emel ridge and the northern slopes of the Zailiysky Alatau to the Ili and Tekes rivers. [1]. The number of the tribe at the end of the 19th century was 100 thousand people [1].

There are two versions of the origin of the tribe. According to the first version, they are part of the Uissun tribe, and Alban is the son of Zharykshak, Baidibek’s grandson. According to the second version, this tribe is related to the Caucasian people of Alban, who lived in the territory of modern Azerbaijan.

The "Uran" of the Alban tribe is Raimbek. Tamga of this tribe is called "Dongelek" [2].


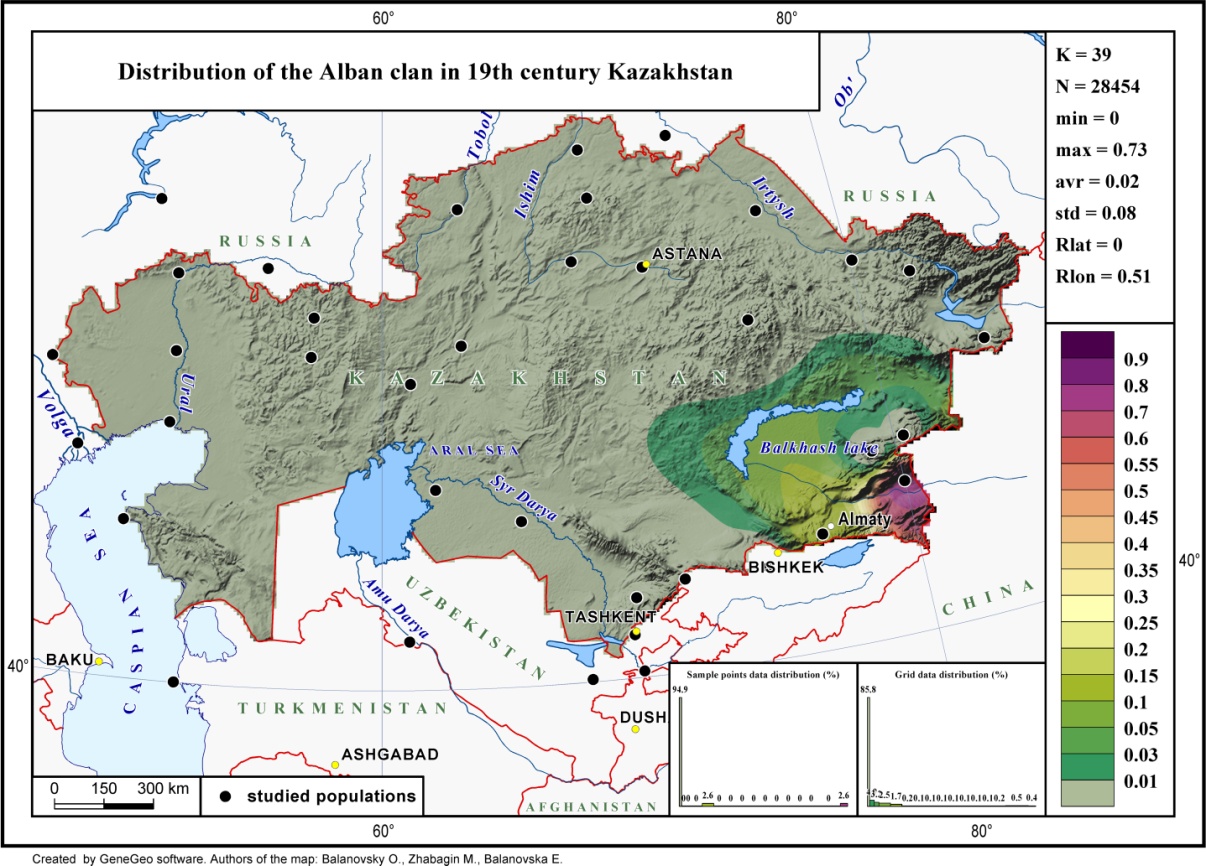


Figure 6. Map of the Alban tribe resettlement [6].

Figure 7. Genealogy of the Alban tribe.

**4. Suan.** The Suan tribe settled in the following territories: foothills of Altyn-Emel and the southeastern slopes of the Dzungarian Alatau, the right bank of the Ili River, from Kokterek in the west to Khorgos in the east [1]. The number of the tribe at the end of the 19th century was 30 thousand people [1].

There are two versions of the origin of the tribe. According to the first version, they are part of the Uissun tribe, and Suan is the son of Zharykshak, the grandson of Baidibek. According to the second version, this tribe is related to the Svans (Georgian subethnos), who lives in the territory of modern Georgia. The "Uran" of the Suan tribe is Baysuan. Tamga of this tribe is called "Dongelek" [2].


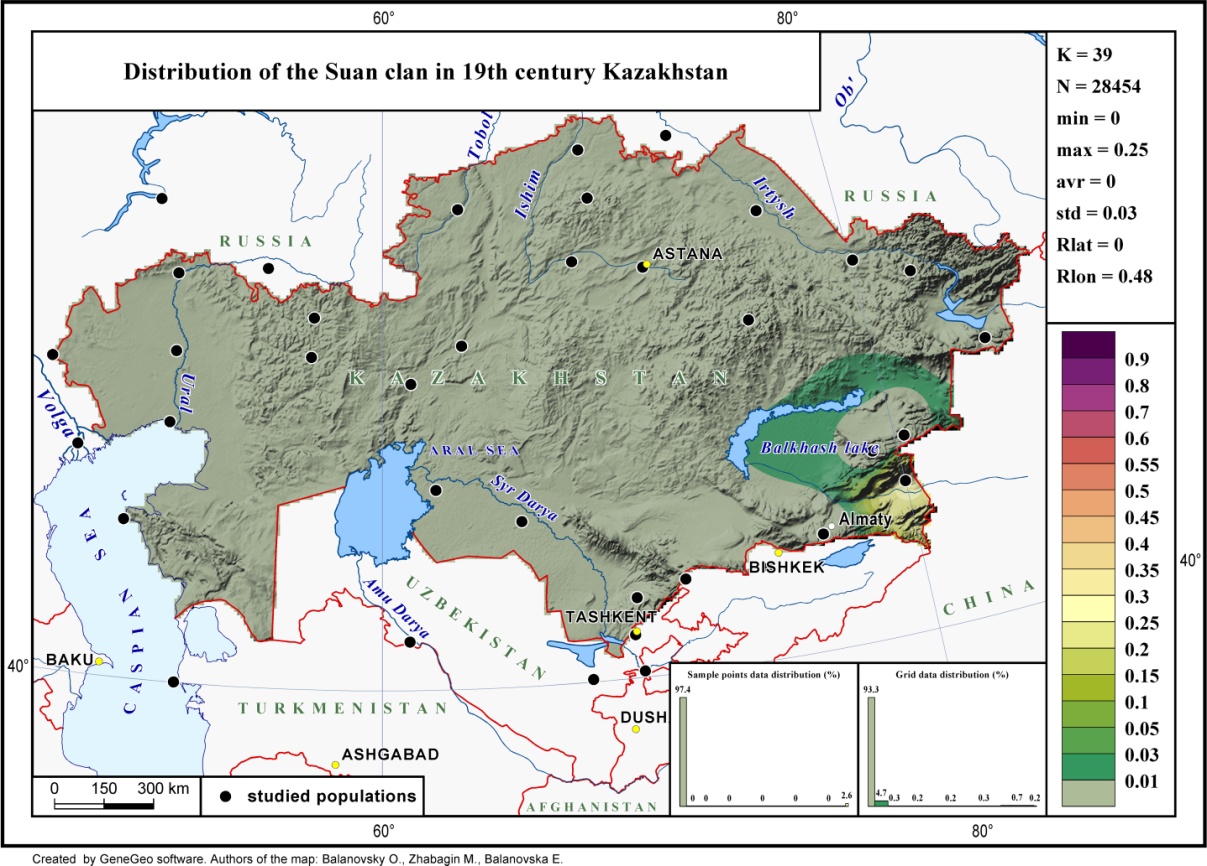


Figure 8. Map of the Suan tribe resettlement [6].

Figure 9. Genealogy of the Suan tribe.

**5. Yssty.** The Yssty tribe settled in the following territories: west part of Zhetysu (Semirechye). The lower reaches of the Ili River, near the southern coast of Lake Balkhash, from the middle course of the Ili River to the foothills of the Zailiysky Alatau, the left bank of the Chu River, the northern and southeastern slopes of the Karatau Mountains, the right bank of the Talas River [1]. The number of the tribe at the end of the 19th century was 40-50 thousand people [1]. The "Uran" of the Yssty tribe is Jauatar. Tamga of this tribe is called "Kosseu" [2].


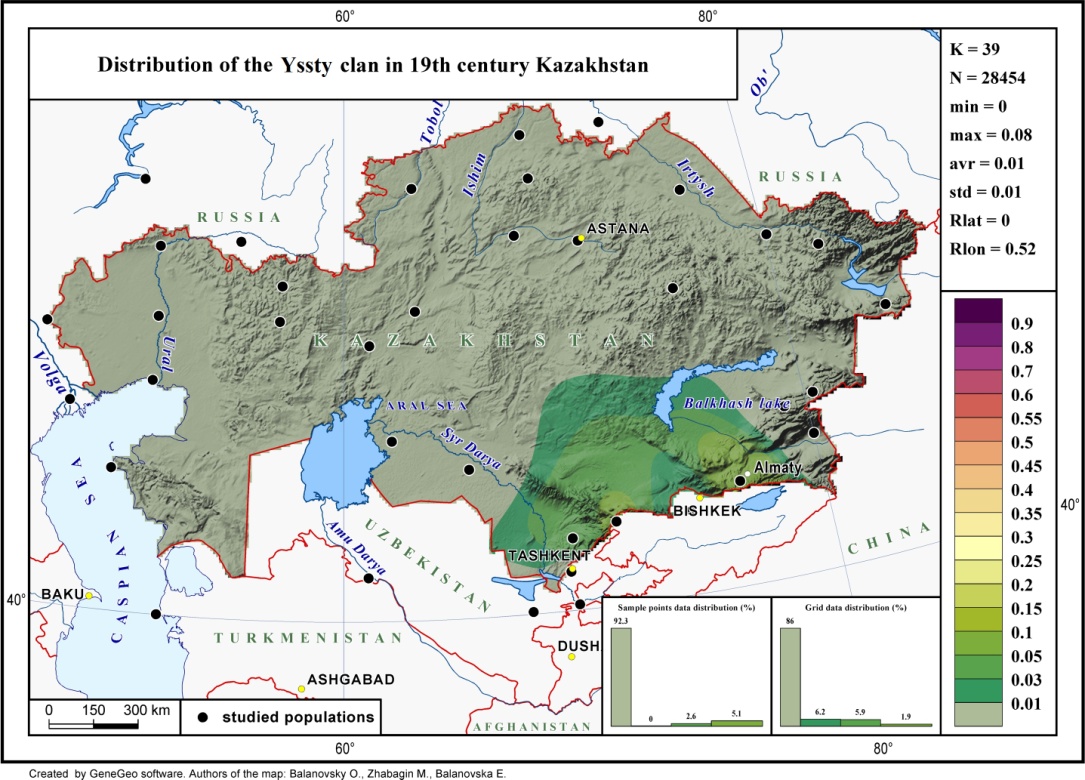


Figure 10. Map of the Yssty tribe resettlement [6].

Figure 11. Genealogy of the Yssty tribe.

**6. Shaprashty.** The Shaprashty tribe settled in the following territories: right bank of the Ili river and its right tributaries, right bank of the Chu river, foothill areas of Zailiysky Alatau [1]. The number of the tribe at the end of the 19th century was 55 thousand people [1]. The "Uran" of the Shaprashty tribe is Karasay. Tamga of this tribe is called "Ay" [2].


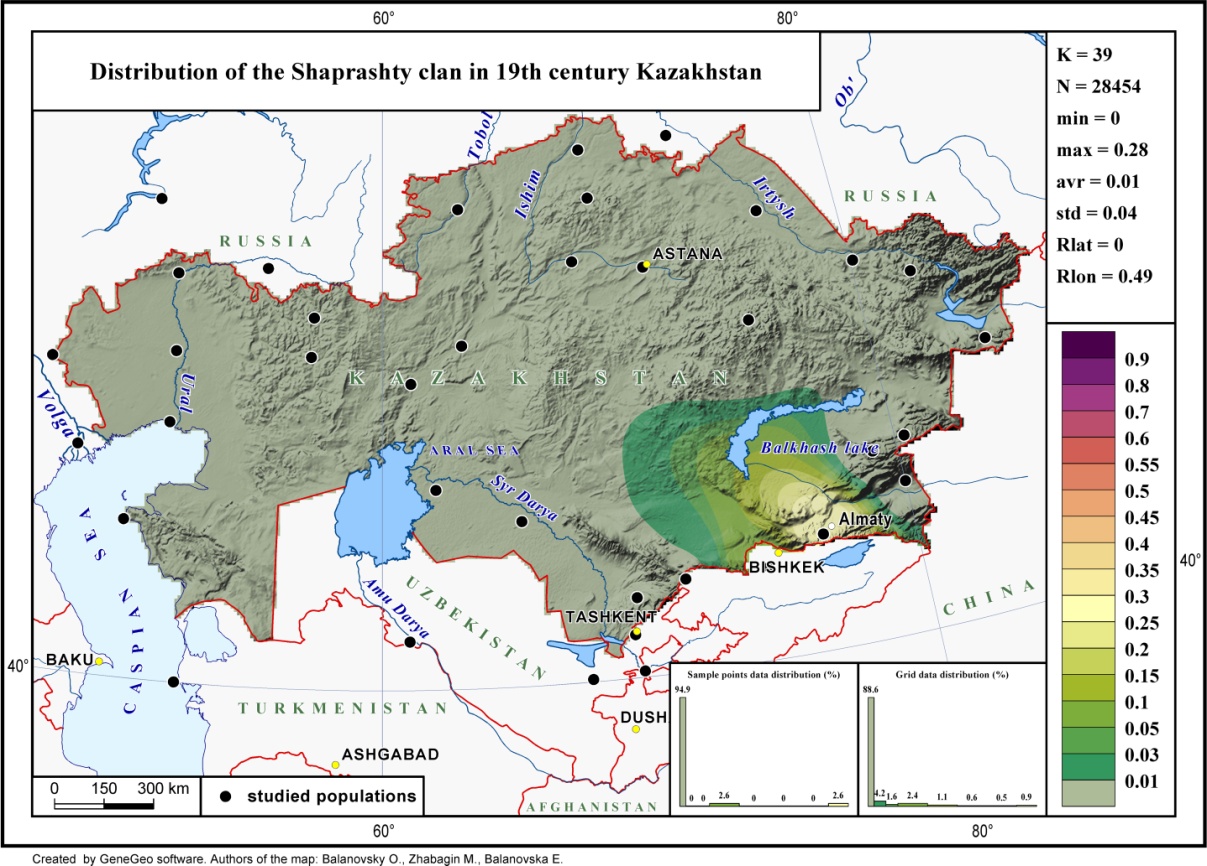


Figure 12. Map of the Shaprashty tribe resettlement [6].

Figure 13. Genealogy of the Shaprashty tribe.

**7. Oshakty.** The Oshakty tribe settled in the following territories: the lower reaches of the Talas River, the southeastern slopes of the Karatau Mountains [1]. The number of the tribe at the end of the 19th century was 15-20 thousand people [1]. The "Uran" of the Oshakty tribe is Bakhtiar. Tamga of this tribe is called "Tumar" [2].


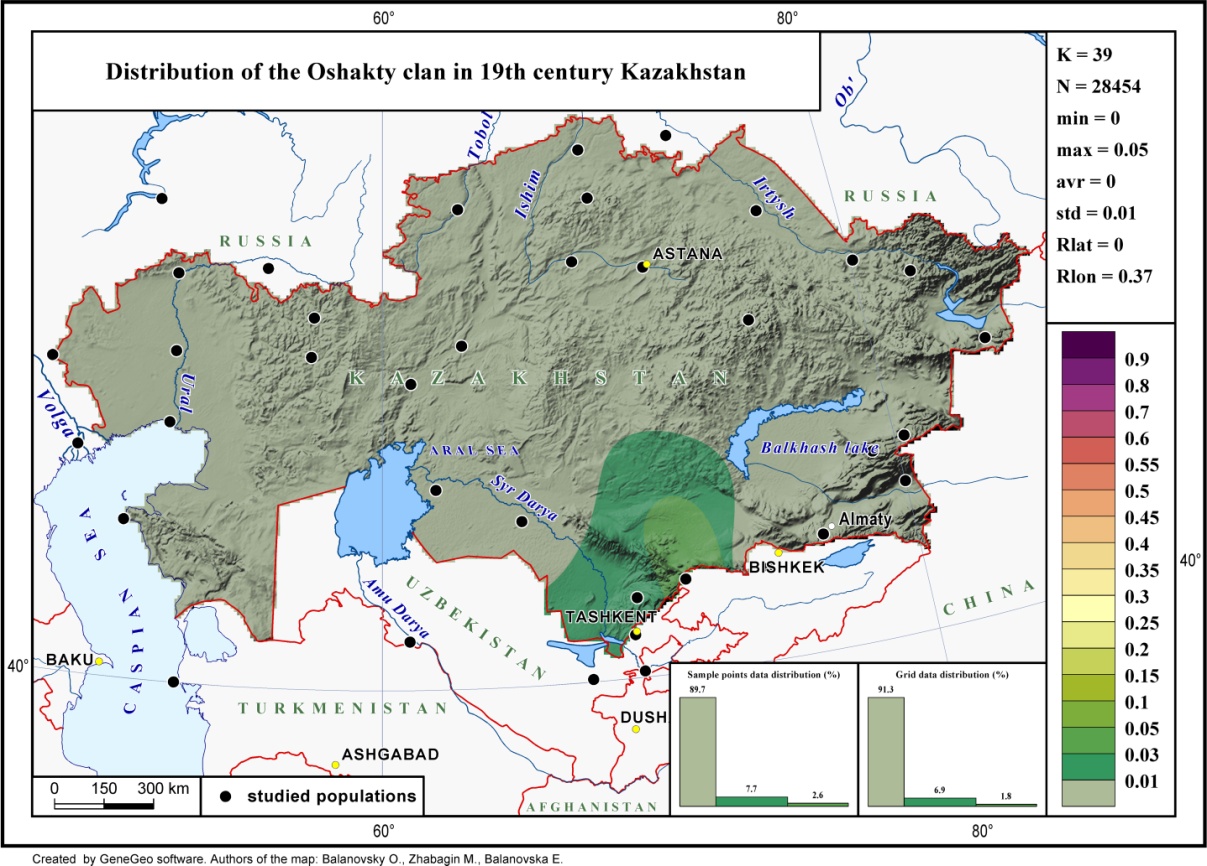


Figure 14. Map of the Oshakty tribe resettlement [6].

Figure 15. Genealogy of the Oshakty tribe.

**8. Shaksham.** The Shaksham tribe settled in in Tashkent and its suburbs. The number of the tribe at the end of the 19th century was unknown [1]. It was smallest tribe of senior zhuz. The "Uran" of the Shaksham tribe is Bakhtiar. Tamga of this tribe is called "Jebe" [2].


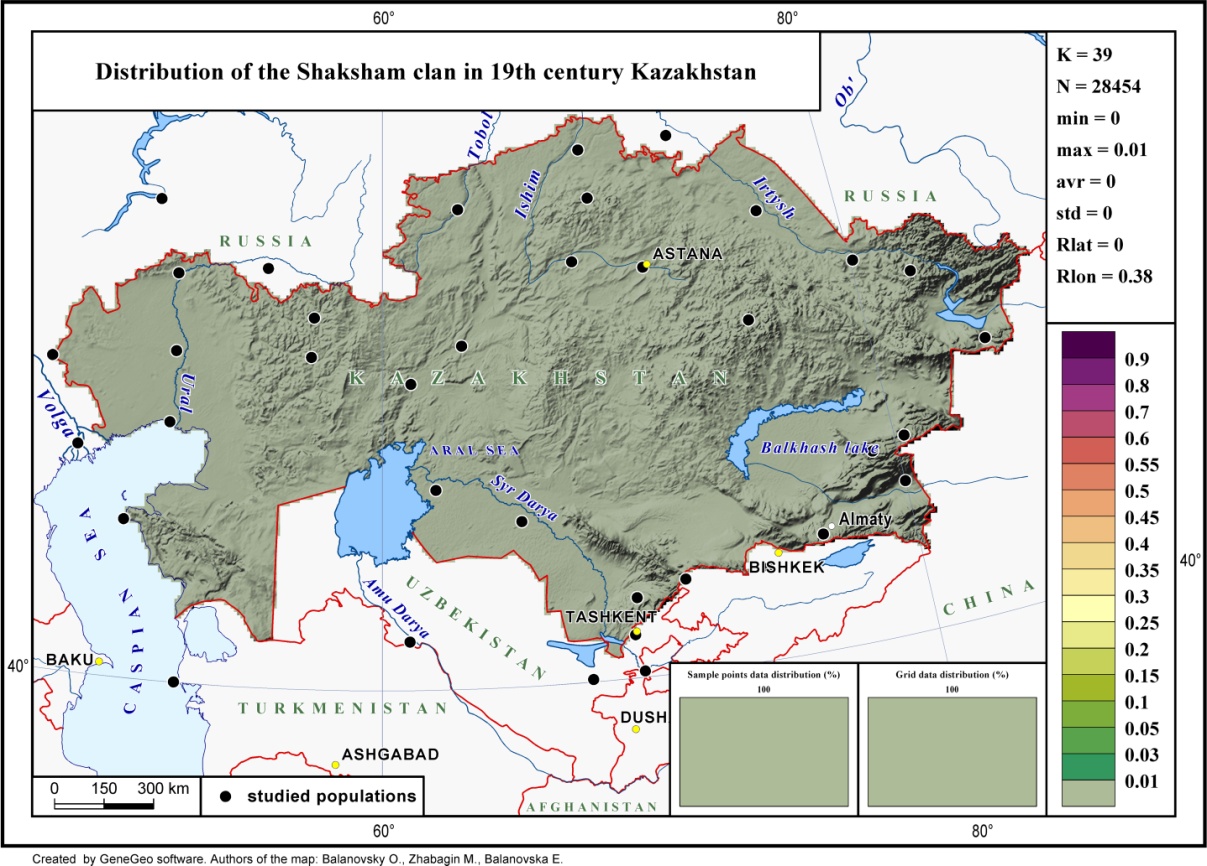


Figure 16. Map of the Shaksham tribe resettlement [6].

Figure 17. Genealogy of the Shaksham tribe.

**9. Syrgeli.** The Syrgeli tribe settled in the following territories: the middle course of the left bank of the Chu River, the lower reaches of the Talas River, the foothills of Karatau [1]. The number of the tribe at the end of the 19th century was 35-40 thousand people [1]. The "Uran" of the Syrgeli tribe is Toganas. Tamga of this tribe is called "Syrge" [2].


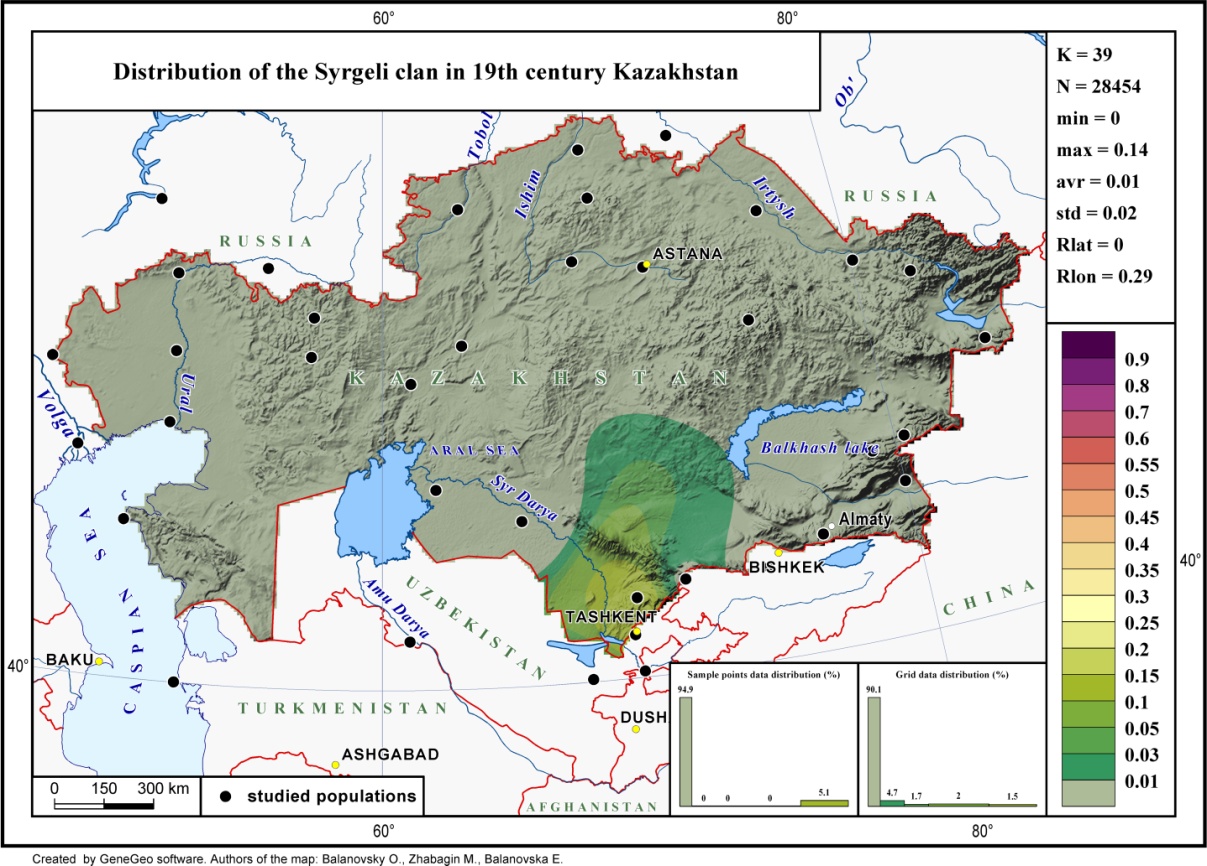


Figure 18. Map of the Syrgeli tribe resettlement [6].

Figure 19. Genealogy of the Syrgeli tribe.

**10. Shanyshkly.** The Shanyshkyly tribe settled in the following territories: south of modern South Kazakhstan region, Tashkent and its suburbs. The number of the tribe at the end of the 19th century was 10 thousand people [1]. According to ethnographic data, this tribe is the descendants of the Katagan екшиу [1], which belonged to the Mongol-Niruns. Representatives of the Katagan tribe live among modern Kazakhs, Uzbeks, Kyrgyz, Hazaras and Mongols. At the beginning of the XVII century, the Katagans accounted for 40% of the number of all Kazakhs, after the civil war in 1627, their number dropped to less than 1%. The "Uran" of the Shanyshkyly tribe is Ayrylmas. Tamga of this tribe is called "Koltanba" [2].


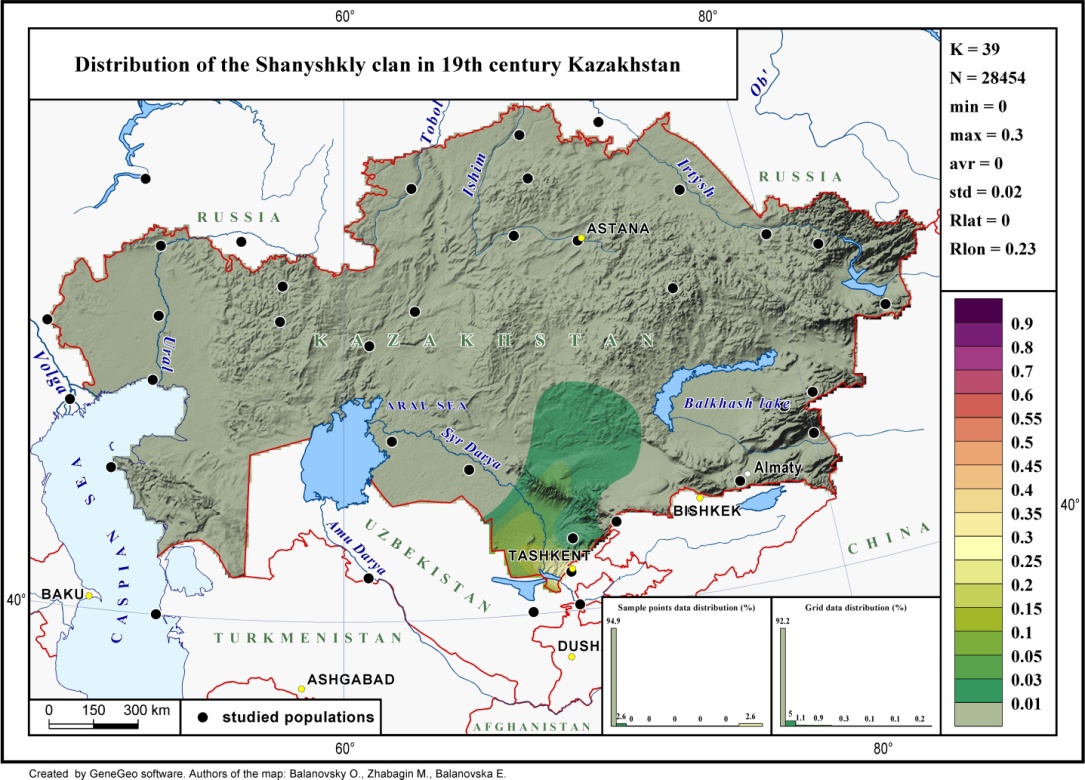


Figure 20. Map of the Shanyshkly tribe resettlement [6].

Figure 21. Genealogy of the Shanyshkyly tribe.

**11. Kanly.** The Kangly tribe settled in the following territories: the left bank of the Ili River and the foothills of Zailiysky Alatau [1]. The number of the tribe at the end of the 19th century was 50 thousand people [1]. According to ethnographic data, this tribe is the descendants of the medieval Turkic people Kangly [1]. Representatives of the Kangly tribe live among modern Kazakhs, Bashkirs, Uzbeks, Kyrgyz, Karakalpaks, Nogais and Mongols of Inner Mongolia. There are several versions of the origin of this tribe. According to the first version, Kangly are descendants of the nomads of the Kangju state. According to the second version, Kangly are descendants of Kangar, which in European sources is known as Pechenegs. The "Uran" of the Kangly tribe is Bayterek. Tamga of this tribe is called "Shylbyr" [2].


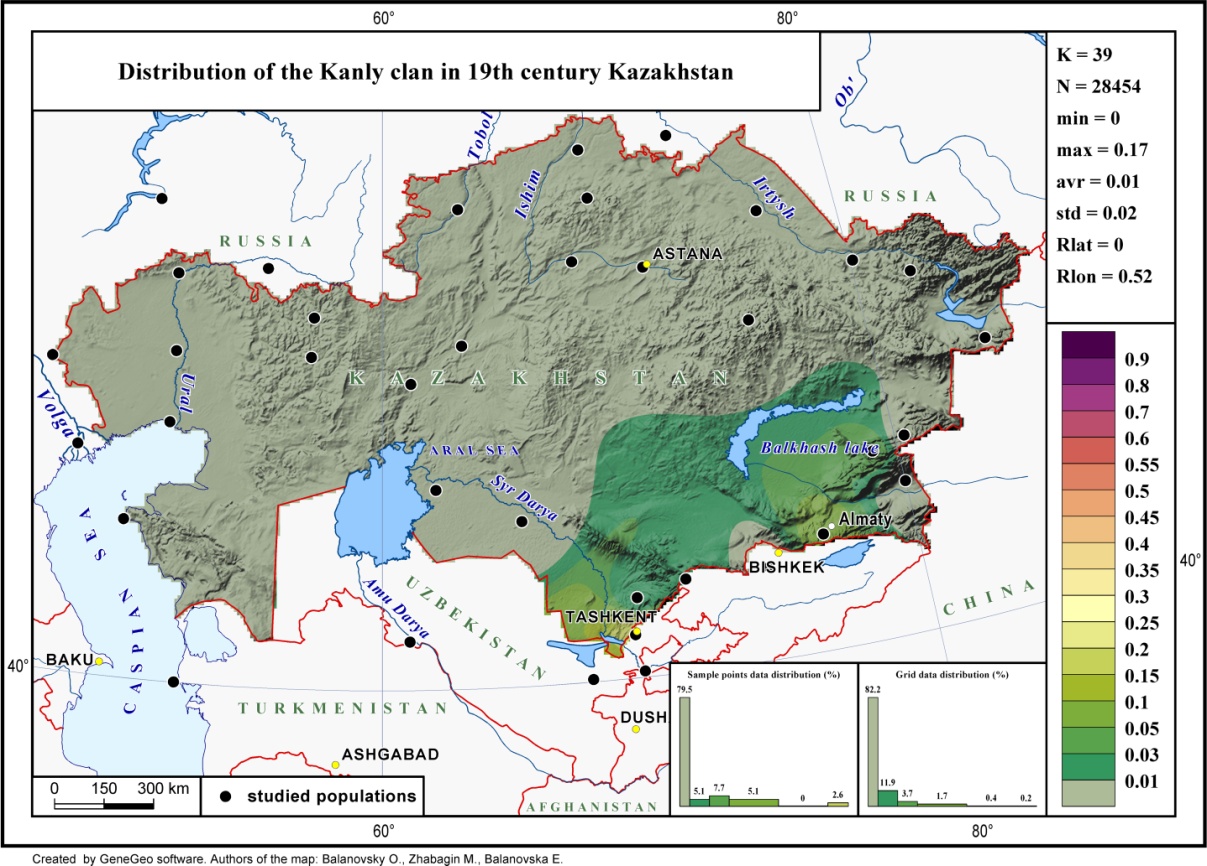


Figure 22. Map of the Kangly tribe resettlement [6].

Figure 23. Genealogy of the Kangly tribe.

**12. Jalair.** The Jalair tribe settled in the following territories: Zhetysu (Semirechye), foothills of the Dzungarian Alatau, Altyn-Emel and Malaysary, between the rivers Ili and Karatal, Balkhash sands, mountains of Arkharly [1]. The number of the tribe at the end of the 19th century was 100 thousand people [1]. According to ethnographic data, this tribe is the descendants of the medieval tribe Jalair, which, according to the most common version, was a descendant of the Yaglakar dynasty, which ruled the Uyghur Kaganate in 745-795. Representatives of the Jalair tribe live among modern Kazakhs, Uzbeks, Hazaras and Mongols. According to one version, the Jalaiirs of the Syrmanak unit are descendants of the Karakalpak Jalairs who joined the Kazakh Jalairs (Shumanak unit) in the 1740s. The "Uran" of the Jalair tribe is Kabylan. Tamga of this tribe is called "Tarak" [2].


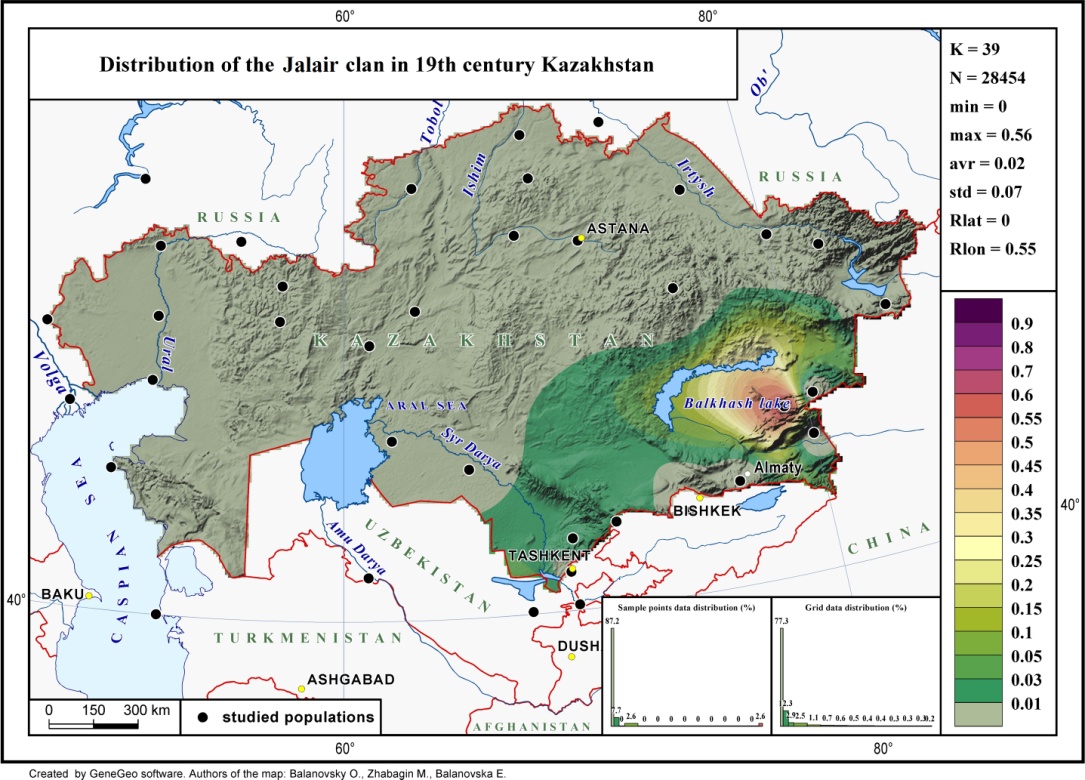


Figure 24. Map of the Jalair tribe resettlement [6].

Figure 25. Genealogy of the Jalair tribe.

**References**

1. *Massanov NE. Nomadic Civilization of Kazakhs: the basics migratory habits of life of society. Almaty: Nurbolat Masanov Fund; 2011. 740 p.*
2. *Beysenbayuly Zh. Qazaq genealogies. Almaty: Ana tіlі; 1994. 160 p.*
3. *Gabzhalilov H. History of Qazaq clans: Dulat. Volume XI. Book 2. Almaty. 2008. 576 p.*
4. *Sadibekov Z. Qazaq genealogies. Tashkent. 1994. 144 p*
5. *Sabitov Zh. Ahmet, son of Aisul (Isa) from the Uissun (Uishin) clan as an epic image // Nogai XXI century. History, Language, Culture. From the sources to the future. Materials of the second international scientific-practical conference. Cherkessk. October 12-13, 2016 Cherkessk. 2016.S. 414-418.*
6. *Zhabagin M.K., Balanovsky О.P., Sabitov Zh.M., Temirgaliyev A.Z., Agdzhoyan A.T., Koshel S.M., Ramankulov Е.М., Balanovska E.V. Reconstructing the genetic structure of the Kazakh from clan distribution data. Vavilovskii Zhurnal Genetiki i Selektsii. 2018;22(7):895-904. doi: 10.18699/VJ18.431*
