# Supplementary material for: The medieval Mongolian roots of Y-chromosomal lineages from South Kazakhstan
Source: BMC Genet. 2020 Oct 22;21(Suppl 1):87. doi: 10.1186/s12863-020-00897-5 (PMC7583311; doi:10.1186/s12863-020-00897-5)
Supplement: Supplementary file 5 — Additional file 5: Figure S1. Genetic relationships of Great zhuz’s clans using Y-SNPs: Multidimensional scaling plot (MDS) [file 12863_2020_897_MOESM5_ESM.pptx]

## Slide 1
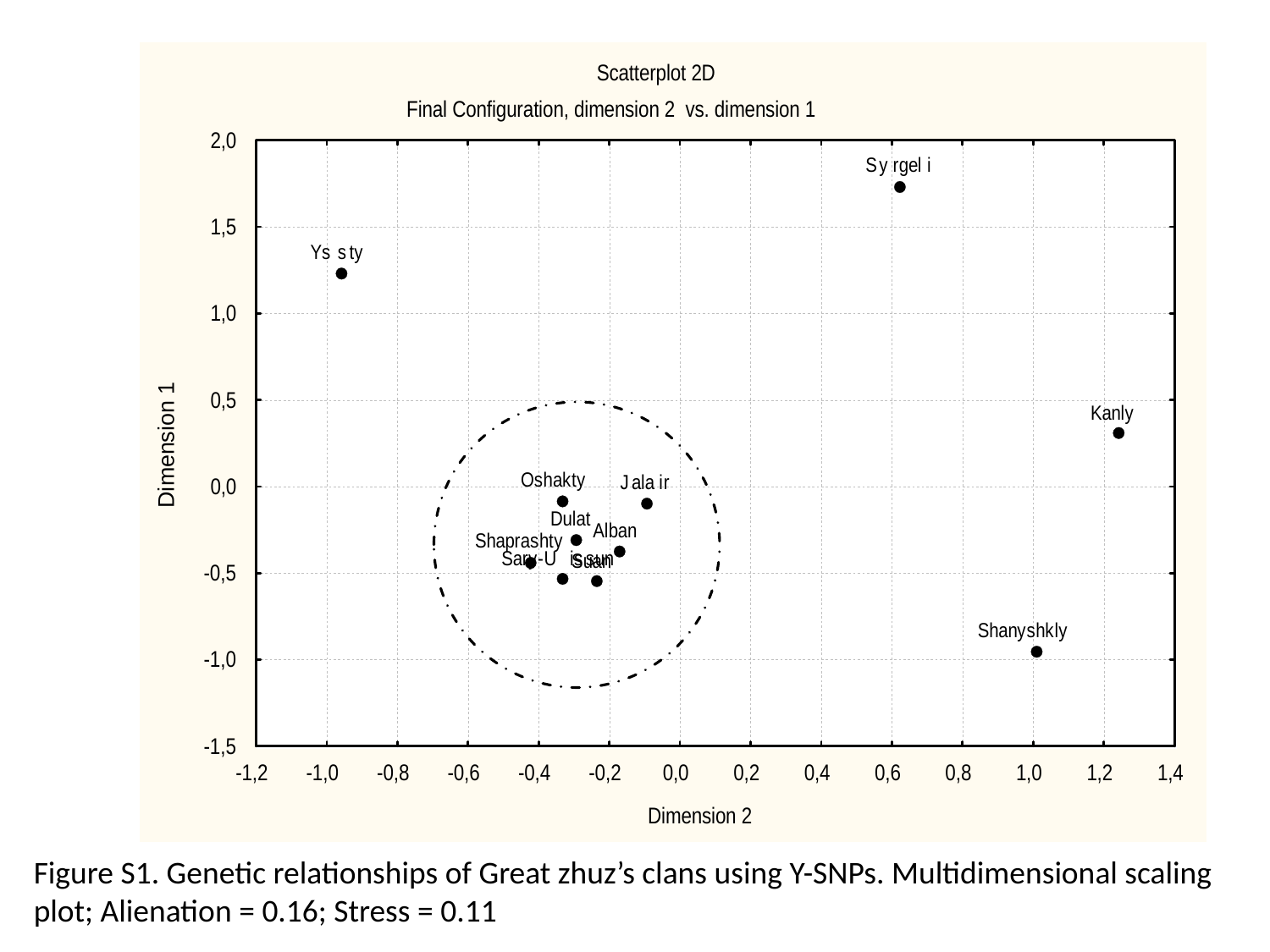

Figure S1. Genetic relationships of Great zhuz’s clans using Y-SNPs. Multidimensional scaling plot; Alienation = 0.16; Stress = 0.11
