# Supplementary material for: The medieval Mongolian roots of Y-chromosomal lineages from South Kazakhstan
Source: BMC Genet. 2020 Oct 22;21(Suppl 1):87. doi: 10.1186/s12863-020-00897-5 (PMC7583311; doi:10.1186/s12863-020-00897-5)
Supplement: Supplementary file 6 — Additional file 6: Figure S2. Genetic relationships of Great zhuz’s clans using Y-SNPs: Principal component analysis (PCA) of the Uissun tribe [file 12863_2020_897_MOESM6_ESM.pptx]

## Slide 1
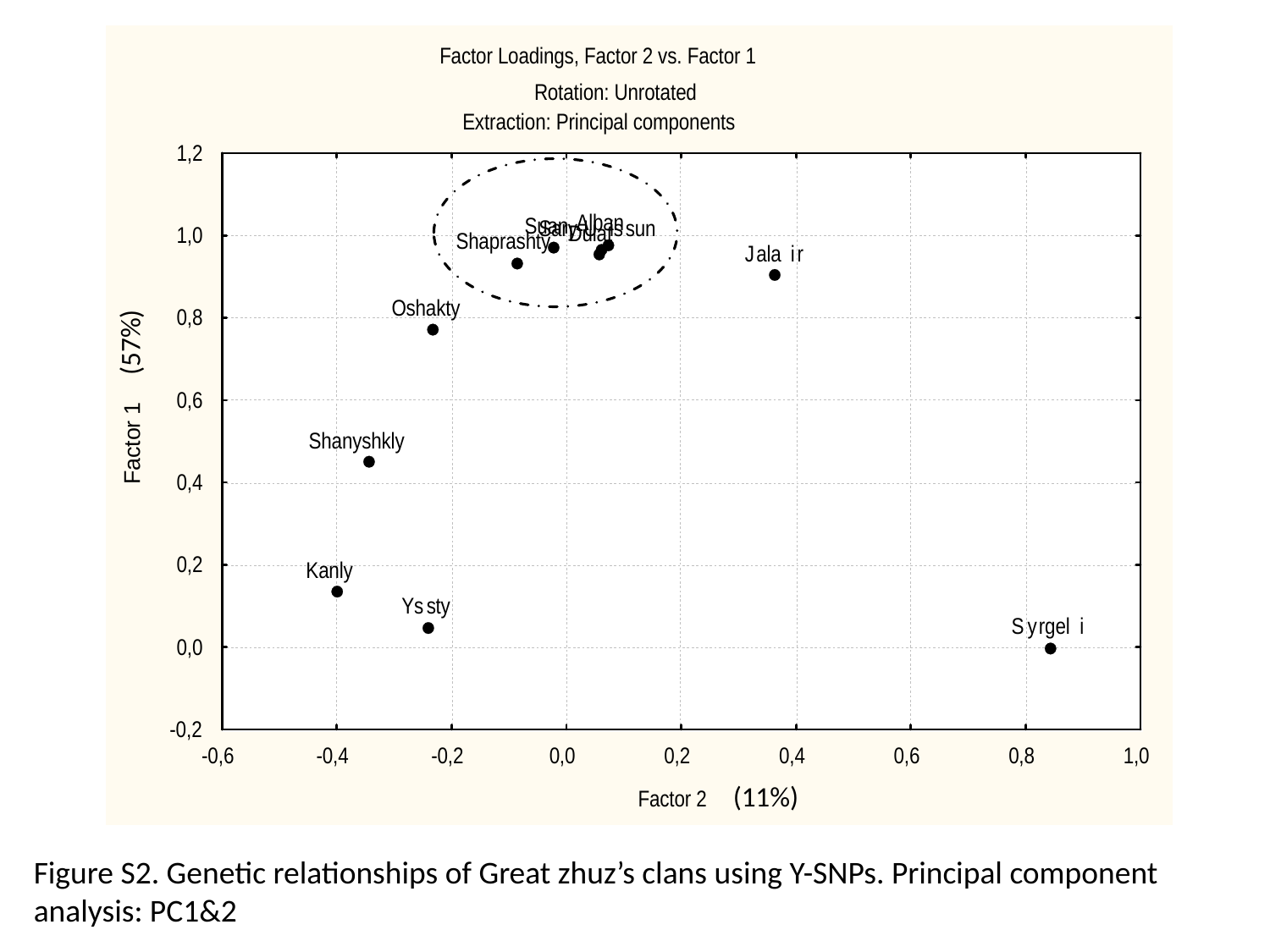

(57%)
(11%)
Figure S2. Genetic relationships of Great zhuz’s clans using Y-SNPs. Principal component analysis: PC1&2
