# Supplementary figures and images for: The medieval Mongolian roots of Y-chromosomal lineages from South Kazakhstan
Source: BMC Genet. 2020 Oct 22;21(Suppl 1):87. doi: 10.1186/s12863-020-00897-5 (PMC7583311; doi:10.1186/s12863-020-00897-5)

## Slide 1
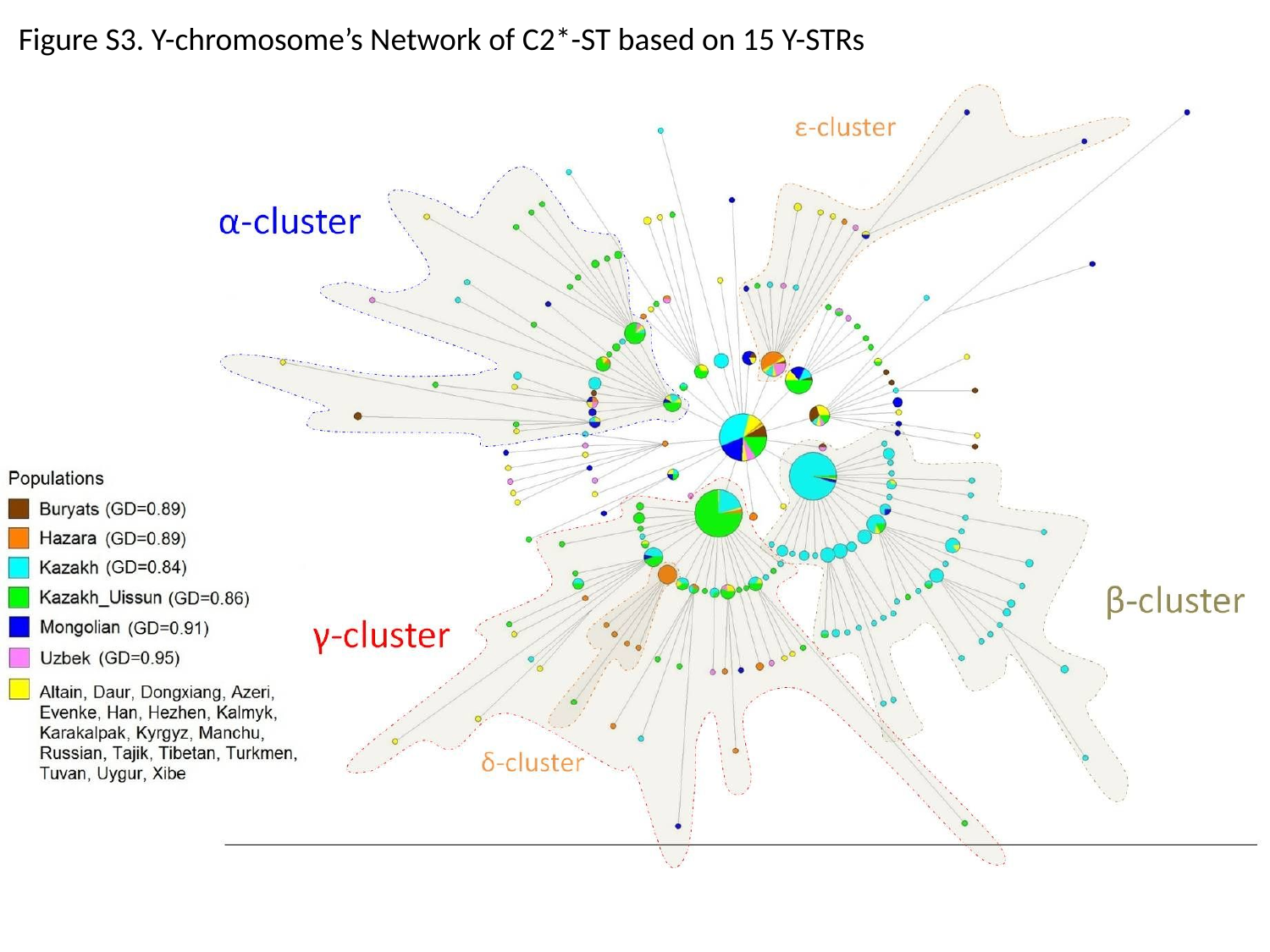

Figure S3. Y-chromosome’s Network of C2*-ST based on 15 Y-STRs

Supplement: Supplementary file 8 — Additional file 8: Figure S3. Y-chromosome’s Network of C2*-ST based on 15 Y-STRs. [file 12863_2020_897_MOESM8_ESM.pptx]

## Slide 1
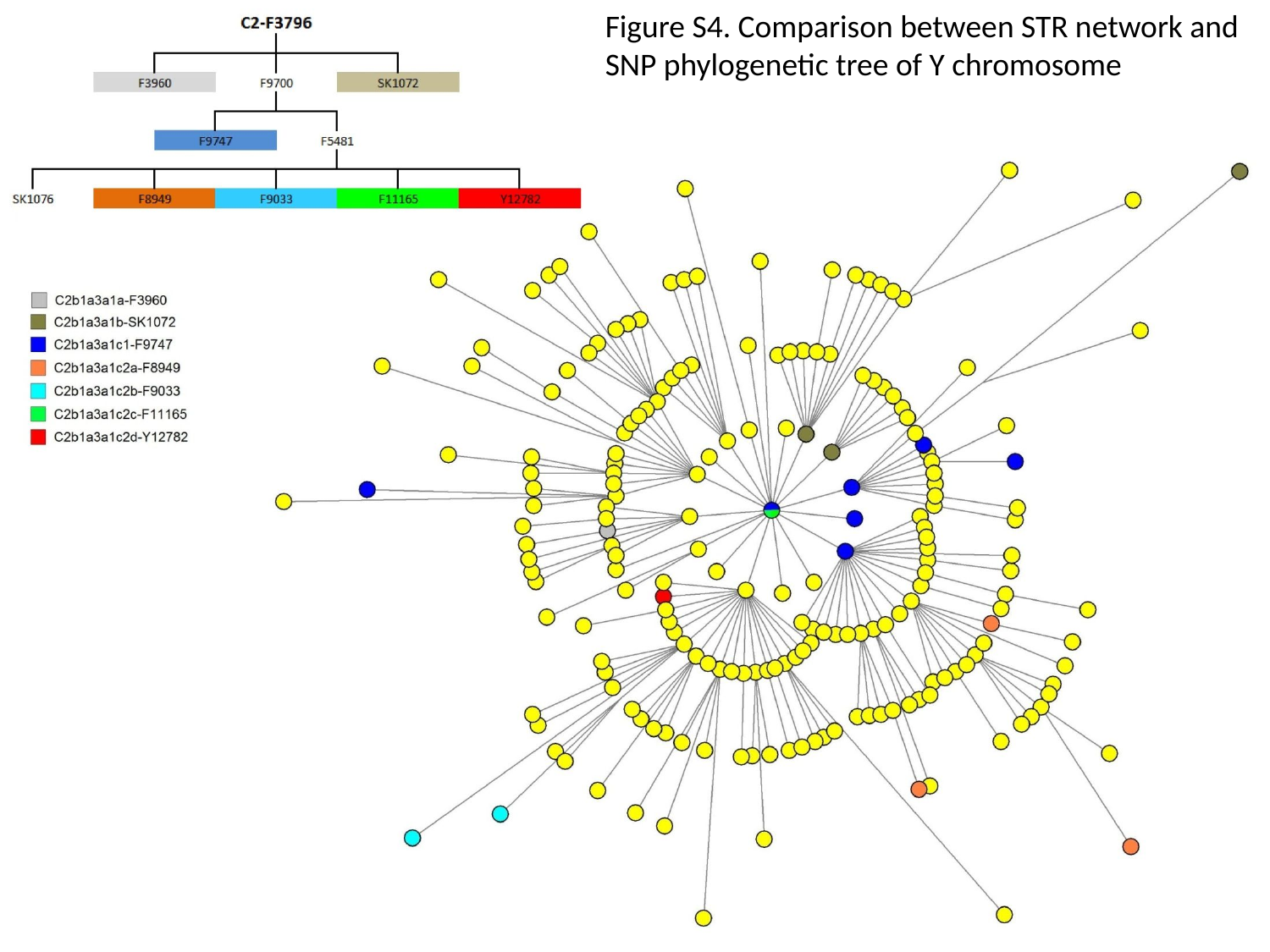

Figure S4. Comparison between STR network and SNP phylogenetic tree of Y chromosome

Supplement: Supplementary file 9 — Additional file 9: Figure S4. Comparison between STR network and SNP phylogenetic tree of the Y chromosome. [file 12863_2020_897_MOESM9_ESM.pptx]

## Slide 1
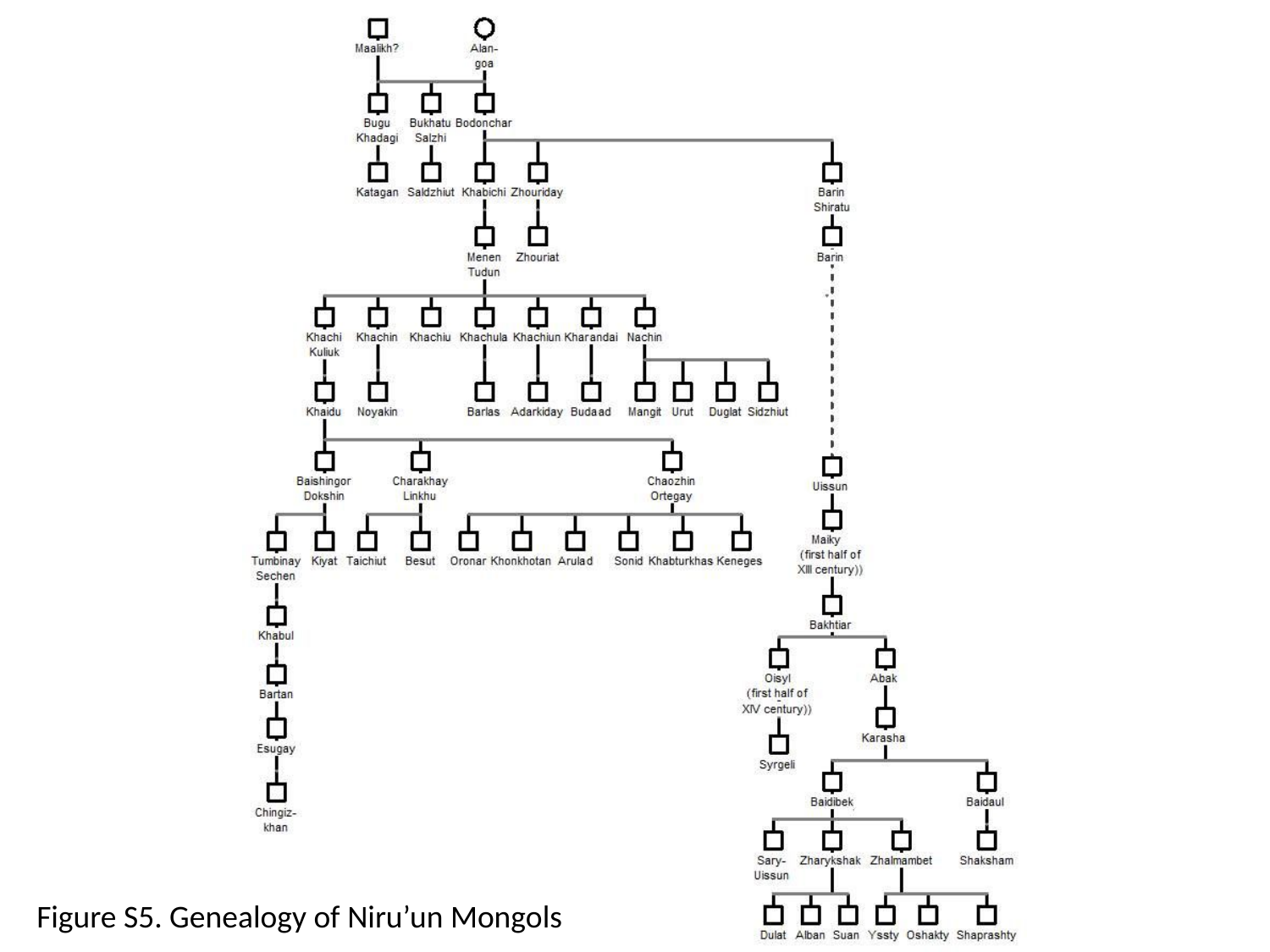

Figure S5. Genealogy of Niru’un Mongols

Supplement: Supplementary file 11 — Additional file 11: Figure S5. Genealogy of the Niru’un Mongols. [file 12863_2020_897_MOESM11_ESM.pptx]
